# Biometry based ageing of nestling Indian Spotted Owlets ( *Athene brama brama*)

**DOI:** 10.3897/zookeys.132.1346

**Published:** 2011-10-03

**Authors:** Satish Pande, Amit Pawashe, Mahadeo N. Mahajan, Anil Mahabal, Reuven Yosef, Neelesh Dahanukar

**Affiliations:** 1 Ela Foundation, C-9, Bhosale Park, Sahakarnagar-2, Pune 411009, Maharashtra, India; 2 Zoological Survey of India, W. R. S. Akurdi, Pune, Maharashtra, India; 3 Ben Gurion University at Eilat, P. O. Box 272, Eilat 88000, Israel. Present address: Women's College for Higher Education, Nagpur, Maharashtra, India

**Keywords:** Spotted Owlet, Age, Biometry, Nestling, Flight-Predictor

## Abstract

Biometric analysis helps in sex differentiation, understanding development and for studies of avian biology such as foraging ecology, evolutionary ecology, and survivorship. We suggest that biometry can also be a reliable, practical and inexpensive tool to determine the age of nestlings in the field by non-invasive methods. As an example we studied the biometry of wing, culmen, talon, tarsus and body mass of nestling southern Indian Spotted Owlets (*Athene brama brama*). Based on the growth pattern analysis using logistic growth model, discriminant analysis and CHAID (Chi-squared Automatic Interaction Detection) based decision tree, we show that biometry of nestling Spotted Owlets is an easy, reliable and inexpensive method to determine nestling age and to assess growth rate and relative nutritional status. These biometric parameters also allow us to predict their ability to initiate first flight from the nest site. This method is described here for the first time and we postulate that such charts can be devised for other avian species as well, so as to assist conservation biologists and bird rescuers.

## Introduction

Adult and juvenile birds of the same species are of similar size but are differentiated on the basis of plumage, fault bars, tail shape, castellated feathers, bill shape, cere color, and other parameters (eg., Svensson 1992; Sutherland 2000). Such differences and others like the appearance of down are less marked and much more subjective in the nestling period, especially if the nestling period is of short duration. Hence, the necessity of some other criteria for determination of the exact age of nestlings is essential. Because body size change is rapid during nestling growth period, biometry can be a useful parameter to determine age since hatching.

Our ability to monitor and understand biometric parameters is important from ecological and conservation perspectives. During the nestling period, these parameters allow one to evaluate parental feeding ability, and to monitor relative nutritional condition between siblings and between neighboring nests of different habitats. Parameters can also help in the rehabilitation of orphaned nestlings in determining whether they are fed appropriately so that growth rates are comparable to nestlings in the wild.

Retrograde calculation of hatching and egg-laying dates can be accurately determined from the age of the nestling or from time of fledging (Blotzheim and Bauer 1980; Cramp and Simmons 1985; Penteriani 2002). However, hatching dates are not always available for nestlings found during field surveys. Biometry has been previously used in the determination of sex of adult owls (Ali and Ripley 1969; Delgadiao and Penteriani 2004), development in owls (Kristan et al. 1996; Nagarajan et al. 2002; Penteriani et al. 2004) and for studies of avian biology such as foraging ecology, evolutionary ecology, and survivorship (Anderson and Norberg 1981; Newton et al. 1983; Clutton-Brock 1986). Biometry is also used to distinguish between subspecies of raptors and other avian species (Ali and Ripley 1969; White and Boyce 1988).

In this paper we show how the use of biometry can be a reliable, practical and inexpensive tool to determine the age of nestlings in the field by non-invasive methods, and by taking the minimum required measurements through the use of a flow chart as described. A method to estimate the time of acquiring flight ability using biometric parameters is also investigated and described. We have taken southern Indian Spotted Owlets (*Athene brama brama*) as an example. Southern Indian Spotted Owlet *Athene brama brama* is a valid subspecies of spotted owlet *Athene brama* and is endemic to this zoogeographical region (Ali and Ripley 1969; Kumar 1985). Feeding and nesting behavior of this species is known (Kumar 1985; Jadhav and Parasharya 2003; Pande et al. 2006), however, detailed biometric analysis of developing nestlings is done for the first time in this study. In this study we present the biometry of the nestling from the day of hatching through fledging.

## Methodology

### Data collection

A total of 53 active nests of southern Indian Spotted Owlet *Athene brama brama* were included in our study. The study was conducted for two consecutive years (2003 and 2004) in and around Saswad (18°19'N to 18°20'N and 73°57'E to 74°01'E) in Pune district, Maharashtra, India. The nests were identified by: a) direct information about known nest sites from local residents, b) passive auditory surveys by authors at dawn and dusk during the breeding season, December to April, when owlets are most vocal, c) searching for probable nest sites, when at least three visits to each site were made, d) by requesting local people to inform about new sites found, after making them familiar about the ongoing study through a public-outreach program.

Out of the 53 active nests found, seven nests were intensively studied during the 2003 breeding season for the documentation of breeding biology and biometry. Biometry of eight nestlings from hatching till fledging, from 0 to 32 days, was done at weekly intervals, and the data was entered serially for each chick. A total of 136 measurements were taken, averaging 17 measurements per nestling. We included only those nests that were easily accessible where the exact dates of egg laying and hatching were recorded. Nestlings that died in the middle of the study were excluded. We ringed each nestling with a numbered aluminum ring placed on the tarsus to facilitate individual identification of each nestling. Sexes are alike in external appearance and thus we could not separate between the nestlings in this study based on their sexes (Hipkis et al. 2002).

We used a Vernier calipers (+0.001mm), wing-stop and tail rulers (0.1mm) for measurement, and Pesola spring scales (60 gm, 100 gm) to weigh body mass. In order to be consistent, only two trained researchers (AP & SP) took all measurements. We measured the biometry of five parameters: (a) wing chord: carpal joint to the tip of the longest primary with the wing flattened, (b) tarsus: ankle joint to the attachment of toes where measurements were taken using flexion at proximal and distal joints, (c) talon: length of claw of the middle toe was measured with Vernier caliper from the point of insertion to the tip, (d) culmen: the exposed part of the culmen from the cere to the tip, (e) body mass: all body mass was taken at sunset. Allowing for the fact that Spotted Owlets forage at dusk or at night, this assured an empty stomach, which minimized the effect of meals. This also caused minimal disturbance to the owlets, which resumed their crepuscular and nocturnal activities immediately after our visits to the nests.

### Statistical analysis

To each of the biometric character we fitted the logistic model to understand its growth pattern and growth rate (Ricklefs 1979). The logistic equation is,

Character value = a/[1+b*exp(-c*Age)] (1)

Where a, b and c are positive constants. Constant *a* signifies maximum possible value of the character, constant *b* signifies the delay in growth associated with the lag phase and constant *c* is the growth rate. The goodness of fit was determined by regression coefficient.

We also studied the growth patterns by nullifying the effect of size using Principle Component Analysis (PCA) as described by Badyaev and Martin (2000). PCA was performed on ln-transformed data of four biometric parameters namely wing chord, talon, tarsus and culmen lengths. The PCA was performed on the covariance matrix. The isometric vector defined as (1/p)^½^, where p is the number of characters, was 0.5. We calculated the angle theta between the eigenvector for each age class and isometric vector to understand the developmental pattern.

We performed Discriminant Factor Analysis (DFA) to understand whether different age groups form significantly different clusters and which factors can best discriminate between the clusters (Legendre and Legendre 1984). We performed Pillay’s trace statistic to find the significant difference between the clusters (Harris 2001).

To predict the nestling age using biometric characters we constructed decision tree (regression tree) using exhaustive CHAID (Chi-squared Automatic Interaction Detection) algorithm. At each step, CHAID chooses the independent (predictor) variable that has the strongest interaction with the dependent variable. Categories of each predictor are merged if they are not significantly different with respect to the dependent variable (Kass 1980).

## Results

### Growth pattern

**Figure 1.**
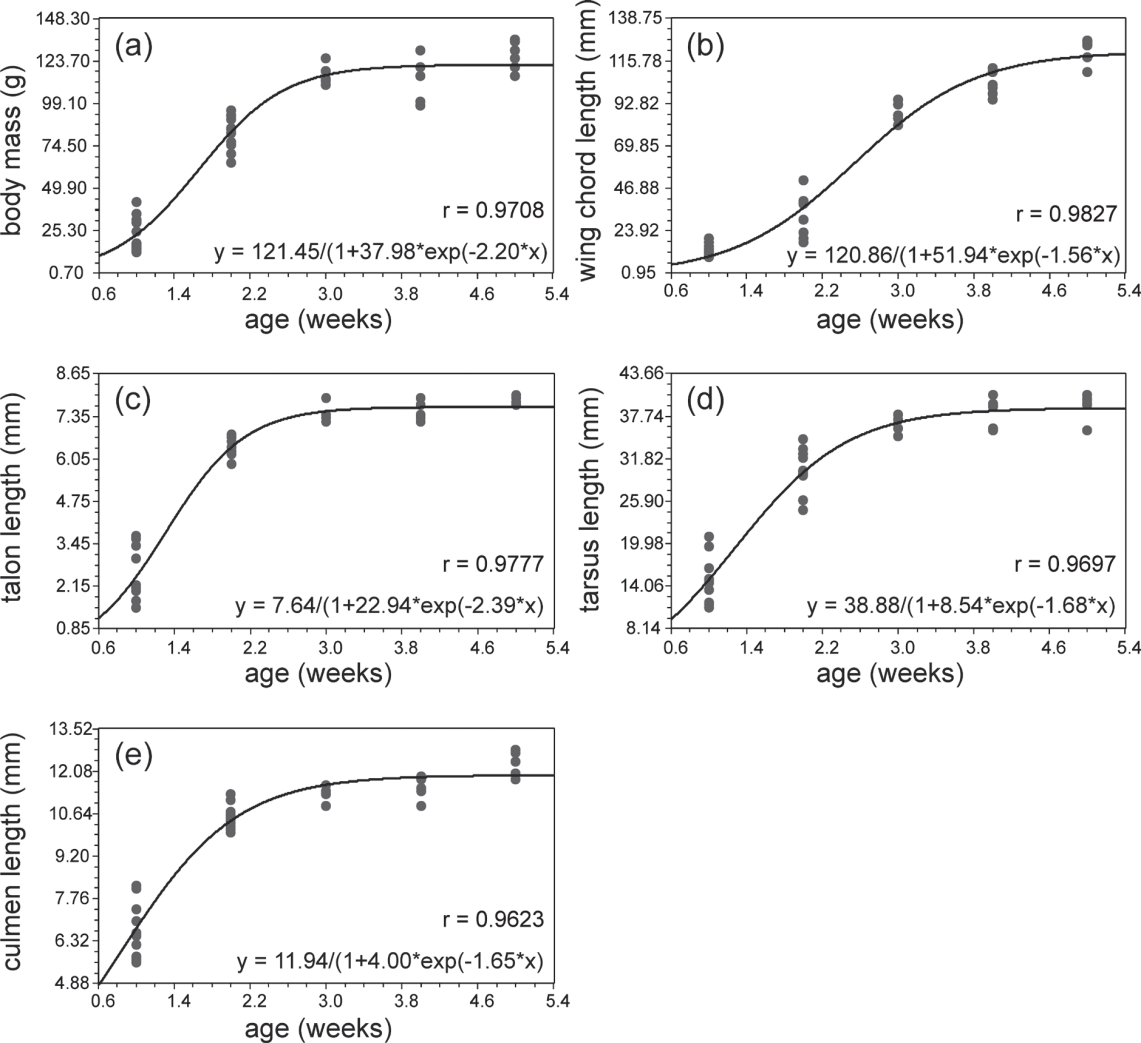
Biometry ofbody mass, wing chord length, talon length, tarsus length and culmen length plotted against the age of nestling Spotted Owlets (*Athene brama brama*). The smoothed curve is the logistic growth model fitted to the data.

**Figure 2.**
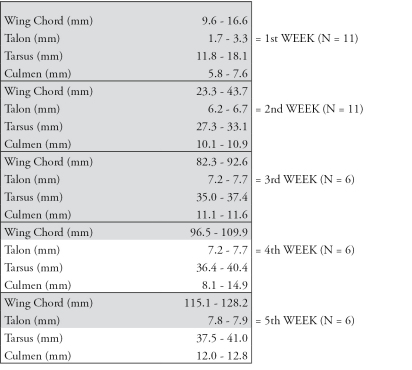
Identification key for ageing nestling Spotted Owlets (*Athene brama brama*) based on biometrics. Highlighted in grey are those parameters that are most reliable for field use.

Observations on the biometric parameters of nestling Spotted Owlets of different age groups are given in Table 1. All biometric characters showed a good fit to logistic model of growth (p < 0.01; Fig. 1). Among the five biometric characters talon length had the highest growth rate (2.39 mm/week) followed by body mass (2.2 g/week), while wing chord length showed the least growth rate (1.56 mm/week). Even though the rate at which both culmen and wing chord increased were similar, wing chord showed initial lag and thus attained a mature chord length at 5 weeks (Fig. 2) while culmen attained maximum length in only two weeks.

**Table 1.** Mean, standard deviation and coefficient of variation of biometric parameters for age in weeks of nestling Spotted Owlets (*Athene brama brama*).

Character	1st Week(n =11)		2nd Week(n =11)		3rd Week(n = 6)		4th Week(n = 6)		5th Week(n = 6)	
	xˉ (sd)	CV	xˉ (sd)	CV	xˉ (sd)	CV	xˉ (sd)	CV	xˉ (sd)	CV
Body Mass (g)	23.36 (9.96)	42.64	82.73 (10.08)	12.19	117.17 (6.62)	5.65	113.83 (12.50)	10.98	126.83 (8.38)	6.60
Wing Chord (mm)	13.06 (3.49)	26.69	33.45 (10.20)	30.48	87.42 (5.14)	5.88	103.17 (6.68)	6.47	121.67 (6.53)	5.37
Talon (mm)	2.46 (0.81)	32.89	6.43 (0.28)	4.29	7.42 (0.25)	3.35	7.48 (0.26)	3.53	7.85 (0.10)	1.34
Tarsus (mm)	14.93 (3.16)	21.18	30.17 (2.91)	9.66	36.22 (1.22)	3.36	38.40 (2.02)	5.26	39.27 (1.76)	4.49
Culmen (mm)	6.74 (0.90)	13.43	10.5 (0.40)	3.86	11.33 (0.23)	2.06	11.57 (3.36)	3.36	12.40 (0.41)	3.35

After a small lag body mass displayed linear growth with a steep rise through 2.5 weeks. Eighty percent of adult growth was attained at the end of 2 weeks (Fig. 1a). At 0–1 week the average mean mass was 26.8 gm (11.1 SD, range 13–42, n = 6) and at 4–5 weeks was 125 gm (7.9, 115–135, n = 5). Coefficient of variation (CoV, 41.46) was highest at one week. The nestlings attained 91.9% of adult body mass at 4.5 weeks, and no significant change was found up to 6.5 weeks. We observed a drop in mass at 3.5 weeks, one week prior to fledging (Table 1).

**Table 2.** Eigenvalues of the first principle component for four ln-transformed biometric characters for eachage class along with percent variability explained by each principle axis and angle theta between eigenvector and isometric vector.

Age	Characters				Variability (%)	theta
	wing	culmen	talon	tarsus		
Week 1	0.5873	0.2099	0.7197	0.3051	76.14423	0.4252
Week 2	0.9869	0.0124	0.0549	0.1515	91.54507	0.9242
Week 3	0.8562	0.0239	-0.4256	0.292	73.92306	1.1886
Week 4	0.6962	0.2667	0.338	-0.5744	87.19698	1.1987
Week 5	0.9299	0.3455	-0.0074	-0.1261	53.56812	0.963

Out of five biometric parameters, wing chord length showed the longest lag phase. It started growing rapidly only after two weeks and attained maximum length after five weeks (Fig. 1b, 2). The average mean value for wing growth at 0–1 week was 13.98mm (3.67, 10.0–20.0, n = 8) and at 4–4.5 weeks 120 mm (7.7, 110–127, n = 4). CoV (35.3) was highest in the second week. This might be due to sex differences in the nestlings, but because of our inability to sex the birds this remains untested. Similar difference during the first week is seen in growth of other parameters also, which accounts for the higher CoVvalues in this period. However, this aspect needs to be evaluated separately in a future study. Wing chord length is 33 % of adult size at two weeks, 80 % of adult size at 4.5 weeks and 85.4 % adult size at 6.5 weeks.

The claw of the middle toe, talon, had the maximum growth rate among the five characters. It showed linear growth up to first two weeks and then it slowed down and attained mature size in the third week (Fig 1c). At 0–1 weeks the average mean value was 2.5 mm (0.84, 2–4, n = 5) and at 4–4.5 weeks was 7.9 mm. Adult size was attained at 4.5 weeks. CoV(33.96) was greatest at 0–1 week (Table 1).

Tarsus showed a steep linear growth up to 2.5 weeks, and adult size was attained at 4.5 weeks (Fig. 1d). At 0–1 week the average mean size was 156 mm (3.5, 110–210, n = 7) and at 4–4.5 weeks was changed to average 389 mm (2.1, 360–410, n = 4). CoV (42.88) was greatest at 1–2 weeks (Table 1).

Culmen had a growth rate equal to the wing chord length however it did not show any lag period and grew rapidly from hatching to the second week (Fig. 1e). Culmen attained adult size in the third week. At 0–1 weeks the average mean size was 6.94 mm (0.96, 6–8, n = 7) and at 4–5 weeks was 12.33 mm (0.5, 12–13, n = 4). CoV (13.8) was highest at 0–1 weeks (Table 1).

In the post-fledging and adult Spotted Owlets showed following biometric characters (n = 8): wing chord 150 mm (145–154), body mass 240 gm (235–245), talon 7.85 mm (7.8–7.9), tarsus 37 mm (33–40), culmen 14.5 mm (14–15)(Ali and Ripley 1969; SP, unpubl. data). One ringed fledgling was recaptured at 6.5 weeks following hatching, 2 weeks after fledging. Its biometrics were: wing chord 126 mm (84% of adult), body mass 125 gm (52.1%), talon 7.9 mm (100%), tarsus 40 mm (108%), and culmen 17 mm (117%). A sibling fledgling of this cohort was also recaptured but only body mass was measured.

We also studied the developmental patterns in the size adjusted characters and compared it with the isometric developmental pattern (Table 2). In the first week of development all other characters showed negative allometric relationship (i.e. relationship less than 1) to talon length (eg. Wing chord length / talon length = 0.5873/0.7197 = 0.82 based on Table 2). For second and third weeks all characters had negative allometric relationship with wing chord length followed by tarsus. For the fourth week all characters had negative allometric relationship with wing chord length followed by talon. While, for fifth week all characters had negative allometric relationship with wing chord length followed by culmen. The angle theta between the eigenvectors and isometric vector increased till fourth week.

### Predicting age from biometric characters

**Figure 3.**
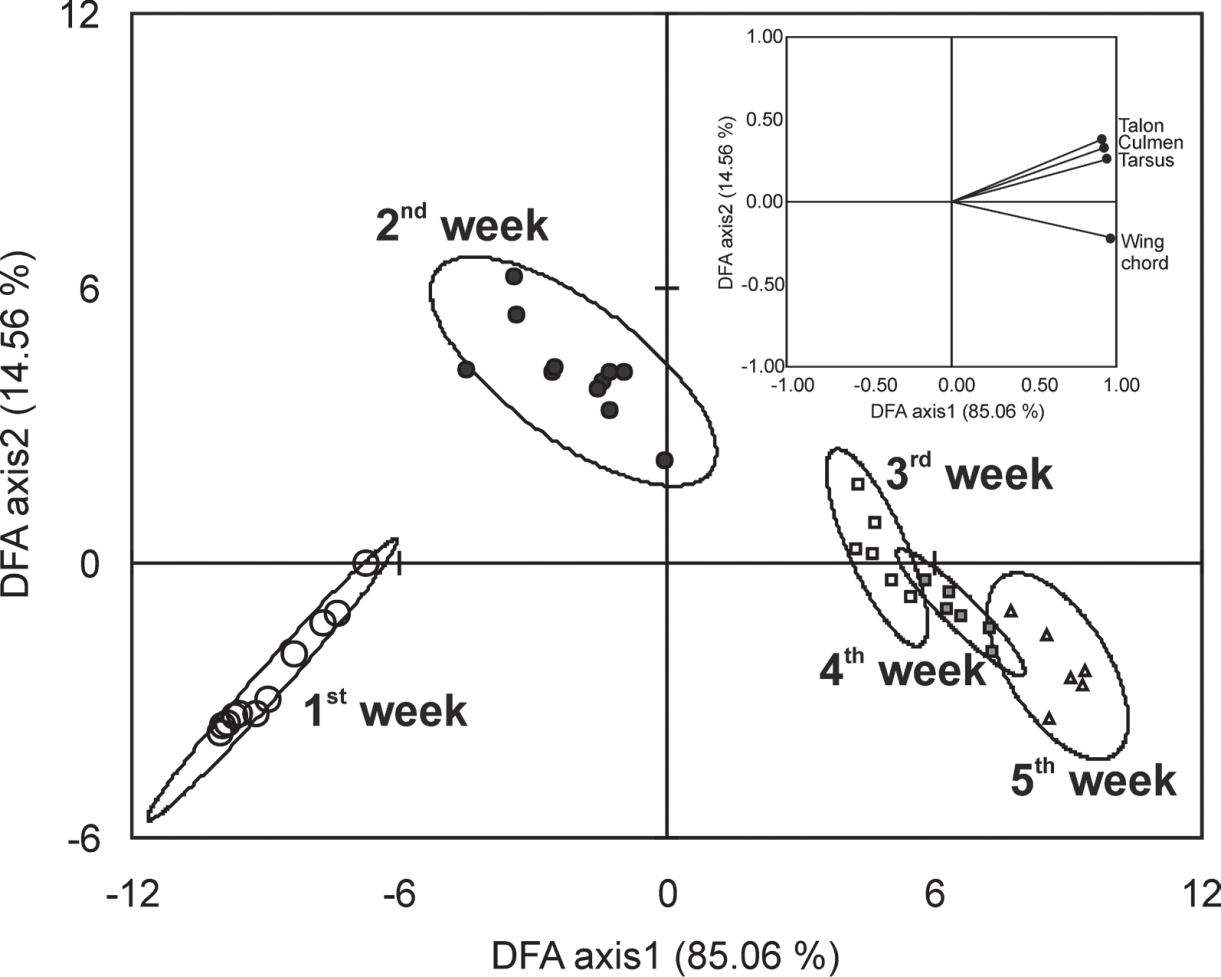
Discriminant Factor Analysis of the age classes based on five biometric characters of nestling Spotted Owlets (*Athene brama brama*). The figure shows the factor scores of observations. Factor loading of variables is given in the inset. Percentages in parenthesis are percent variation explained by each factor.

Growth rate of different parameters varied significantly with respect to age. This allowed us to derive useful biometric parameters to predict age during the nestling period (Fig. 2). The Discriminant Factor Analysis (DFA) of the data extracted four factors but only first two factors had eigenvalues more than one. The first factor explained 84.86% of the total variation in the data while the second factor explained 14.46% of the total variation and together they explained 99.32% of the variation. All five age groups formed significantly different clusters in DFA (Pillay’s trace = 2.211, F = 8.408, p < 0.0001). Clusters of the first two weeks showed significantly separate clusters while clusters of remaining weeks had partial overlaps (Fig. 3). All factors had high factor loading on the first factor but the maximum was for wing chord length and tarsus length (Fig. 3). Fig. 3 also depicts that first week nestlings can be differentiated from second week nestlings with the biometric characters talon length, culmen length and tarsus length while the nestlings of the second, third, forth and fifth weeks can be primarily separated from each other only using the wing chord length. These observations were consistent with the PCA analysis (Table 2).

**Figure 4.**
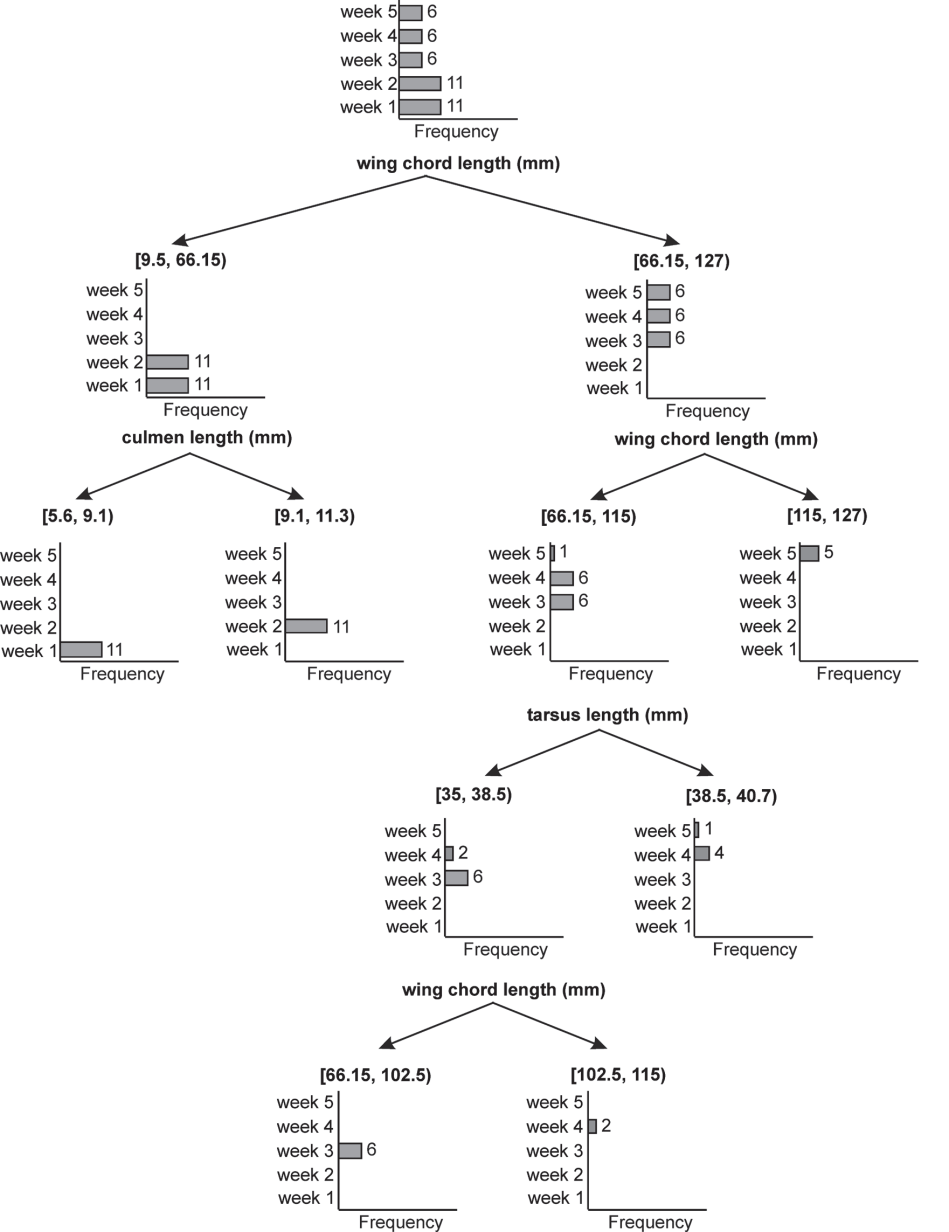
Decision tree based on exhaustive CHAID algorithm. Bar diagrams show the number of individuals in each age class 1 to 5 weeks of nestling Spotted Owlets (*Athene brama brama*). Numbers on the bars indicate individuals in the given age class.

To predict the age of a nestling from the minimum biometric characters, we constructed a regression tree using CHAID algorithm. The regression tree could separate the nestling of different ages using three characters - wing chord length, culmen length and tarsus length (Fig. 4). The decision rules which separate the nestling of different ages according to Fig. 4 are as follows.

If wing chord length (mm) is in the range [9.5, 66.15] then Age = 1 week in 50% of cases.

If wing chord length (mm) is in the range [66.15, 127] then Age = 3 weeks in 33.3% of cases.

If culmen length (mm) is in the range [5.6, 9.1] and wing chord length (mm) is in the range [9.5, 66.15] then Age = 1 week in 100% of cases.

If culmen length (mm) is in the range [9.1, 11.3] and wing chord length (mm) is in the range [9.5, 66.15] then Age = 2 weeks in 100% of cases.

If wing chord length (mm) is in the range [66.15, 115] then Age = 3 weeks in 46.2% of cases.

If wing chord length (mm) is in the range [115, 127] then Age = 5 weeks in 100% of cases.

If tarsus length (mm) is in the range [35, 38.5] and wing chord length (mm) is in the range [66.15, 115] then Age = 3 in 75% of cases.

If tarsus length (mm) is in the range in [38.5, 40.7] and wing chord length (mm) is in the range [66.15, 115] then Age = 4 weeks in 80% of cases.

If wing chord length (mm) is in the range [66.15, 102.5] and tarsus length (mm) is in the range [35, 38.5] then Age = 3 weeks in 100% of cases.

If wing chord length (mm) is in the range [102.5, 115] and tarsus is in the range [35, 38.5] then Age = 4 weeks in 100% of cases.

However, we also present a simple flow-chart style of the biometrics with the minimum-maximum measurements (Fig. 2). In this case all five parameters were important to identify the age of the nestling during the first three weeks post-hatching. But only wing chord length was a reliable parameter for the whole study period, especially during the fourth and fifth weeks. Talon growth in the 3rd and 4th week was similar but greater in the 5th week. Tarsus and culmen were reliable only for the first three weeks of post-hatching growth.

### Predicting capacity of flight

Correlation between wing chord size and body mass was examined in order to understand when the fledglings are capable of flight. Examination of biometric data of mass gain and wing chord growth revealed that in the early nestling period the growth rate of wing size was less than that of mass, but was equal at 4.5 weeks.

Based on the above, we devised a formula to examine this correlation and determine the optimal wing chord length to body mass ratio to predict when the nestling would be capable of initiating its first attempt at flight.

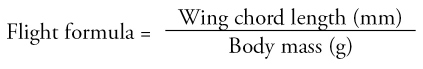



**Figure 5.**
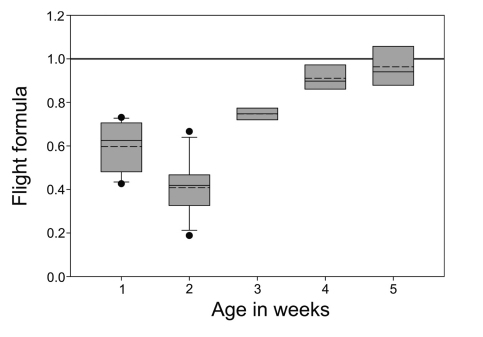
Flight formula for different age groups of nestling Spotted Owlets (*Athene brama brama*). Box plot of flight formula index are plotted for different age groups. When the flight formula approaches unity birds are ready to fly.

The average value of flight formula of fifth week nestling was 0.96 while nestlings of younger age showed lesser values (Fig. 5). Nestlings of age 1^st^ to 4^th^ week showed average flight formula values 0.59, 0.41, 0.75 and 0.91 respectively. Adults had a test value of 0.97 for males (n = 7) and 0.97 for females (n = 6). However, two fledglings that were flying well at 6.5 weeks showed a test value of 0.99.

## Discussion

In this study we have used only externally measured biometric parameters in order to understand nestling growth rate and to present an idea of how we can use these data to help future studies that may need to evaluate age of hatchlings in Spotted Owlets. However, we consider our limited sources to also be a limiting factor in the depth to which this study could be done at present. Our inability to sex individuals at the nest prevents us from knowing the differential growth rates of males compared to females. Ali and Ripley (1981) mention that females are larger than males but do not explain how to separate the sexes. It is a well known phenomenon that in owls the sex ratio of offspring is adjusted to food availability (cf. Appleby et al. 1997; Sasvari and Nishiumi 2005). Future studies should avail of techniques such that we can not only present a general picture for the subspecies but also of the sex-specific differences in biometrics and their growth rates.

In our study, all five biometric parameters studied in Spotted Owlets showed logistic growth during the nestling period from hatching till 4.5 weeks when they fledged. The growth rate of all the parameters examined in this study is differential, this heterochrony is used to estimate the age of the nestlings. Three parameters - talon, tarsus and culmen - achieve 100 % growth at 4.5 weeks, while body mass gain is 89% and wing chord growth is 80% of adults at 4.5 weeks.

We attribute this differential growth to the required capabilities that enable fledging. Upon fledging each of the individuals has to fend for itself – from predators and for food. This requires fully developed talons, culmen and tarsus, and a significant increase in body mass. In many cases we have observed Spotted Owlets nestlings - similar to many other owl species - walk out of the nest hole and perch on branches of the nest tree prior to gaining the ability of flight. We assume that the reason for this is to escape increased risk of predation at the nest, where the stench and odor from accumulated pellets, fecal matter and other debris is likely to attract predators. We base this assumption on our having documented predation of eggs and nestlings from the nest hole by Small Indian Civet (*Viverricula indica*) and House Crows (*Corvus splendens*). We realize that predation avoidance is only one of many reasons, and it is obvious that it’s impossible to learn to fly while housed inside a small, crowded nest cavity.

Owing to the fact that the mean values of wing chord, tarsus and body mass differed significantly between the age groups, based on the flow chart, a comparison of the field measurement with our data will allow the researcher to estimate age with a high level of accuracy. We suggest that only three characters (tarsus length, culmen length and wing chord length) are sufficient for determining the age of the nestling.

The estimation of nestling age from this flow chart is useful for conservation and rescue work. The biometric parameters obtained can be compared with the values of various parameters plotted against age in normal wild nestlings given in this paper. From this normal base line trend, one may evaluate the growth and assess the nutritional status of nestlings reared in an orphanage or by foster parents. If a discrepancy is apparent, appropriate corrective measures can be taken by adjustments in feeding.

The nestlings attained 91.9% of adult body mass at 4.5 weeks but experienced a drop in mass at 3.5 weeks, one week prior to fledging. This trend is also recorded in other birds of prey towards the end of nestling stage and can be explained as a response to achieve appropriate wing loading in order to make the first flight easier (Brown 1976).

An analysis of the wing chord length and the body mass using our flight formula (eq. 2) suggests that as the nestling approaches maturity and is capable of first attempt of flight the flight formula value approached unity. In the simplest words our analysis shows that as the wing chord length (mm) approaches body mass (g) the bird becomes capable of flight. This finding is important for rescued fledglings that have suffered injuries or fractures, and for which, appropriate time of release needs to be calculated. The optimal body mass to wing chord ratio helps decide the amount of feeding and time required between rescue and release. We have observed that hand-reared nestlings and rehabilitated adult owls may exhibit good wing flapping, but do not immediately take flight when released. Hence, with the application of our formula one can predict the capability of flight and thus prevent predation after release.

In summary, biometry of wing, body mass, talon, tarsus and culmen of nestling Spotted Owlets is an easy, reliable and inexpensive method to determine nestling age, to assess growth rate and nutritional status, and to predict ability to initiate first flight. This method is described here for the first time for Spotted Owlets, and we postulate that such charts can be devised for other avian species as well.

## References

[B1] Ali S Ripley SD (1981) Handbook of the birds of India and Pakistan together with those of Bangladesh, Nepal, Bhutan and Sri Lanka. Vol. 3. New Oxford University Press, Delhi.

[B2] Anderson M Norberg RA (1981) Evolution of reversed sexual size dimorphism and role partitioning among predatory birds, with a size scaling of flight performance. Biological Journal of Linnaean Society 15: 105-130. 10.1111/j.1095-8312.1981.tb00752.x

[B3] Appleby BM Petty SJ Blakey JK Rainey P Macdonald DW (1997) Does variation of sex ratio enhance reproductive success of offspring in Tawny Owl (*Strix aluco*)? Proceedings of the Royal Society of London B 264: 1111–1116. 10.1098/rspb.1997.0153

[B4] Badyaev AV Martin TE (2000) Individual variation in growth trajectories: phenotypic and genetic correlations in ontogeny of the house finch (*Carpodacus mexicanus*). Journal of Evolutionary Biology 13: 290–301. doi; 10.1046/j.1420-9101.2000.00172.x

[B5] Brown L (1976) Birds of prey: their biology and ecology. A & W Publishers, New York.

[B6] Clutton-Brock TH (1986) Sex ratio variation in birds. Ibis 128: 317-329. 10.1111/j.1474-919X.1986.tb02682.x

[B7] Cramp S Simmons KEL (1985) Handbook of the birds of Europe, the Middle East and North Africa IV. Oxford University Press, Oxford.

[B8] Kristan DM Qutierrez RJ Franklin AB (1996) Adaptive significance of growth patterns in juvenile spotted owls. Canadian Journal of Zoology 74: 1882-1886. 10.1139/z96-212

[B9] Delgado MM Penteriani V (2004) Gender determination of Eurasian Eagle-Owls (*Bubo bubo*) by morphology. Journal of Raptor Research 38: 375-377.

[B10] Desfor KB Boomsma JJ Sunde P (2007) Tawny Owls *Strix aluco* with reliable food supply produce male-biased broods. Ibis 149: 98-105.

[B11] Harris RJ (2001) A primer for multivariate statistics. Third Edition. Lawrence Erlbaum Associates Publishers, London.

[B12] Hipkiss T Hornfeldt B Eklund U Berlin S (2002) Year-dependent sex-biased mortality in supplementary-fed Tengmalm’s owl nestlings. Journal of Animal Ecology 71: 693-699. 10.1046/j.1365-2656.2002.t01-1-00635.x

[B13] Jadhav A Parasharya BM (2003) Some observations on the nesting behaviour and food of the spotted owlet *Athene brama*. Zoos’ Print Journal 18: 1163-1165

[B14] Kass GV (1980) An exploratory technique for investigating large quantities of categorical data. Applied Statistics 29: 119-127. 10.2307/2986296

[B15] Kumar TS (1985) The life history of the Spotted Owlet (*Athene brama brama*, Temminck) in Andhra Pradesh. Raptor Research Centre, Hyderabad, India.

[B16] Legendre P Legendre L (1984) Numerical ecology. Second edition. Elsevier Sciences, Amsterdam.

[B17] Nagarajan R Thiyagesan K Natarajan R Kanakasabai R (2002) Patterns of growth in nestling Indian Barn-Owls. Condor 104: 885-890. 10.1650/0010-5422(2002)104[0885:POGINI]2.0.CO;2

[B18] Newton I Marquiss M Rothery P (1983) Age structure and survival in a sparrow hawk population. Journal of Animal Ecology 52: 591-602. 10.2307/4574

[B19] Pande SA Pawashe M Mahajan N Mahabal A (2006) Changing nest site preference for holes in earth cuttings in Spotted Owlet *Athene brama*. Indian Birds 2: 7-8

[B20] Penteriani V Delgado MM Maggio C Aradis A Sergio DF (2005) Development of chicks and predispersal behaviour of young in the Eagle Owl *Bubo bubo*. Ibis 147: 155-168. 10.1111/j.1474-919x.2004.00381.x

[B21] Ricklefs RE (1979) Patterns of growth in birds. V. A comparative study of development in the starling, common tern, and Japanese quail. Auk 96: 10-30.

[B22] Sasvari L Nishiumi I (2005) Environmental conditions affect offspring sex-ratio variation and adult survival in Tawny Owls. Condor 107: 321-326. 10.1650/7621

[B23] Sutherland W (2000) The Conservation Handbook. Research, Management and Policy. Blackwell Science. 10.1002/9780470999356

[B24] Svensson L (1992) Identification Guide to European Passerines. Naturhistorika Riksmuseet, Stockholm.

[B25] White CM Boyce DA Jr. (1988) An overview of Peregrine Falcon subspecies. In: Cade TJ Enderson JH Thelander CG White CM (Eds). Peregrine Falcon Populations: Their Management and Recovery. Braun-Brumfield, San Francisco: 789-810.

